# The Landscape and Therapeutic Targeting of *BRCA1*, *BRCA2* and Other DNA Damage Response Genes in Pancreatic Cancer

**DOI:** 10.3390/cimb45030135

**Published:** 2023-03-03

**Authors:** Ioannis A. Voutsadakis, Antonia Digklia

**Affiliations:** 1 Division of Medical Oncology, Department of Internal Medicine, Sault Area Hospital, Sault Ste. Marie, ON P6B 0A8, Canada; 2 Division of Clinical Sciences, Northern Ontario School of Medicine, Sudbury, ON P3E 2C6, Canada; 3 Department of Oncology, Centre Hospitalier Universitaire Vaudois, 1011 Lausannne, Switzerland

**Keywords:** DNA damage response, homologous recombination, pancreatic adenocarcinoma, PARP, ATR inhibitors

## Abstract

Genes participating in the cellular response to damaged DNA have an important function to protect genetic information from alterations due to extrinsic and intrinsic cellular insults. In cancer cells, alterations in these genes are a source of genetic instability, which is advantageous for cancer progression by providing background for adaptation to adverse environments and attack by the immune system. Mutations in *BRCA1* and *BRCA2* genes have been known for decades to predispose to familial breast and ovarian cancers, and, more recently, prostate and pancreatic cancers have been added to the constellation of cancers that show increased prevalence in these families. Cancers associated with these genetic syndromes are currently treated with PARP inhibitors based on the exquisite sensitivity of cells lacking *BRCA1* or *BRCA2* function to inhibition of the PARP enzyme. In contrast, the sensitivity of pancreatic cancers with somatic BRCA1 and BRCA2 mutations and with mutations in other homologous recombination (HR) repair genes to PARP inhibitors is less established and the subject of ongoing investigations. This paper reviews the prevalence of pancreatic cancers with HR gene defects and treatment of pancreatic cancer patients with defects in HR with PARP inhibitors and other drugs in development that target these molecular defects.

## 1. Introduction

Pancreatic cancer is a prevalent malignancy, second only after colorectal cancer among digestive system cancers [1]. An estimated 62,210 new cases of pancreatic adenocarcinoma will be diagnosed in the United States in 2022 and almost 50,000 deaths are expected from the disease [1]. Pancreatic cancers represent 3% of all cancers in both men and women, but they are responsible for 8% of all cancer deaths in both sexes. Globally, the prevalence of pancreatic adenocarcinoma was more than 495,000 cases and the death toll was more than 465,000 in 2020 [2]. Projections show that prevalence and mortality will increase in the coming decades [3]. Pancreatic cancer tends to be diagnosed in advanced stage, and this, together with its distinct treatment resistance, results in a 5-year survival rate of less than 5% and a median survival of 6 months.

In recent years, elucidation of the molecular pathogenesis of pancreatic cancer and molecular alterations contributing to its development and propagation has ignited hopes for therapeutic translation that would improve disease outcomes [4,5]. The molecular pathology of pancreatic adenocarcinomas is characterized by a paucity of prevalent mutations that can be targeted therapeutically [6]. Few cancer-associated genes display recurrent mutations in pancreatic cancers. Mutations in oncogene *KRAS* are the most prevalent alterations and occur in 80% to 90% of pancreatic adenocarcinomas. Mutations in gene *TP53* encoding for tumor suppressor p53 are also very prevalent, occurring in about 70% of cases. Neither of these alterations are currently therapeutically targeted, except for *KRAS* G12C mutations, which can be targeted with kinase inhibitors sotorasib and adagrasib but are rare in pancreatic cancer [7]. In addition, mutations in two other tumor suppressor genes, *CDKN2A*, encoding for cell cycle inhibitor p16, and *SMAD4*, both with prevalence exceeding 10% in pancreatic cancer, are not currently therapeutically targeted.

Mutations in breast- and ovarian-cancer-predisposing genes *BRCA1* and, especially, *BRCA2* are also associated with increased risk for pancreatic cancer development [8,9]. These genes are mutated in lower frequencies than the above-mentioned four pancreatic- cancer-associated genes but are of particular therapeutic interest because they can be targeted with PARP inhibitors [10]. The protein products of these genes are involved in DNA damage response and repair of double-strand DNA breaks through homologous recombination [11]. Mutations in *BRCA1* and *BRCA2* lead to homologous recombination deficiency (HRD) and cause sensitivity to drugs that inhibit enzyme PARP (poly-ADP ribose polymerase). Germline *BRCA1* and *BRCA2* mutations have been associated with benefit from PARP inhibitors in pancreatic cancer, and inhibitor olaparib has been approved for maintenance treatment of metastatic pancreatic cancer patients with such mutations after non-progression to first-line platinum-based chemotherapy [12]. In contrast, a benefit from PARP inhibitors in pancreatic cancer in other settings, such as somatic *BRCA1*/*BRCA2* mutations, and in pancreatic cancers with mutations in non-*BRCA1*/*BRCA2* DNA damage response (DDR)-associated genes is less clear. Ongoing HRD is a prerequisite for PARP inhibition sensitivity and may be produced by several molecular defects of proteins involved in the process. However, the degree of HRD produced by each individual defect is variable and may be dependent on redundancy of HR protein functions and presence of alternative pathways that bypass the defect. In addition, germline and somatic defects have different implications, the former being stably present in all the cells of the patient while the latter developing in clones of cancer cells with variable allele frequency. This paper will examine the landscape of DDR-associated gene mutations in pancreatic adenocarcinomas and therapeutic development of drugs targeting the process, with a focus on cancers with both germline and somatic alterations in *BRCA1* and *BRCA2* and cases with mutations in DDR genes other than *BRCA1* and *BRCA2*.

## 2. The Landscape of DDR Mutations in Pancreatic Cancer

Four publicly available genomic series of pancreatic adenocarcinoma patients, published from The Cancer Genome Atlas (TCGA), the MSK-IMPACT study, the pancreatic cancer sub-cohort of a pan-cancer study from China and the pancreatic cancer cohort from the American Association for Cancer Research (AACR) project GENIE (public v.13), were interrogated [6,13,14,15]. Analysis of the series of interest was performed in cBioportal for cancer genomics (www.cbioportal.org (accessed on 12 December 2022)), a genomic studies platform that is freely accessible and allows for interrogation of genomic alterations in any gene of interest included in the original studies [16]. The OncoKB precision oncology knowledge base was used to derive the functional significance of identified mutations [17]. OncoKB classifies mutations of cancer-associated genes as oncogenic/likely oncogenic or alternatively as likely neutral or of unknown significance.

In the pancreatic TCGA cohort of 184 patients, 42 cases (22.8%) have alterations in one or more of an extended panel of DDR-related genes (Table 1) [6]. Most frequent alterations are amplifications that are encountered in 29 cases (15.8%), with a total number of 46 amplifications (some cases possess amplifications in more than one of these genes). A total of 25 mutations in DDR-related genes are encountered in 15 cases (8.2%) of the pancreatic TCGA cohort. Nine of these mutations (4 in *ATM*, 2 in *BRCA2*, 2 in *ATR* and 1 in *RAD51C*) are considered oncogenic or likely oncogenic. Thus, seven cases (3.8%) in the TCGA pancreatic cohort possess (likely) oncogenic mutations in non-*BRCA1*/*BRCA2* DDR-related genes.

In the pancreatic adenocarcinoma patients from the MSK-IMPACT cohort, alterations in DDR-related genes were present in 46 of 383 cases (12%) [13]. Twenty cases (5.2%) contained oncogenic or likely oncogenic mutations in these genes (Table 1). Ten of those (2.6% of the cohort) are in *BRCA2* and two cases were in *BRCA1*. One additional case had a mutation in the other core DDR gene, *PALB2*. The second most commonly mutated DDR gene after *BRCA2* was *ATM,* with 5 mutated cases in the MSK-IMPACT pancreatic cancer cohort [13]. Three additional genes, *BARD1*, *BRIP1* and *CDK12,* possessed mutations in two, one and one case, respectively (Table 1).

In the pancreatic sub-set of the Chinese OrigiMed cohort that included 461 analyzed patients, DDR-related genes were altered in 62 cases (13%), with most alterations described being mutations [14]. Oncogenic or likely oncogenic mutations were present in 30 patients (6.5%) of the cohort. Most frequent oncogenic mutations were in *ATM* gene, which were present in 16 cases (3.5%), followed by *BRCA2* in 5 cases and *PALB2* and *ATR* with 3 mutated cases each. *BRCA1*, *RAD51D*, *XRCC2* and *MRE11* showed 1 oncogenic or likely oncogenic mutation each (Table 2).

In the pancreatic adenocarcinoma cases of the project GENIE, which included 5262 cases profiled, the most prevalent oncogenic or likely oncogenic mutations in DDR-related genes were in *ATM* and in *BRCA2*, followed by lower frequencies in *BRCA1*, *PALB2*, *ATR* and *BRIP1* and a few cases in other genes (Table 2) [15].

A cohort of 2818 pancreatic ductal adenocarcinomas evaluated with the Caris Life Sciences (Phoenix, AZ, USA) genomic panel disclosed a prevalence of *BRCA1* mutations in 1.3% of cases, of *BRCA2* mutations in 3.1% of cases and of *PALB2* mutations in 0.6% of cases [18]. Cases with *BRCA1*, *BRCA2* and *PALB2* mutations had a lower prevalence of *TP53* mutations than wild type cases. This study examined also biomarkers of immune response in *BRCA1-*, *BRCA2-* and *PALB2*-mutated pancreatic cancers and showed that *BRCA1-* and *BRCA2-* but not *PALB2*-mutated cancers had a higher prevalence of PD-L1-positive staining (defined as staining of >1% cancer cells in immunohistochemistry) than wild type counterparts. A higher mean tumor mutation burden (TMB) and higher prevalence of microsatellite instability were also observed in cases with mutations in the three genes [18].

It is clinically important to note that a minority of pancreatic cancer patients with germline mutations in *BRCA1*/*BRCA2* and other DDR genes have no family history of cancers [19]. Thus, guidelines currently advocate for universal genetic testing in all pancreatic cancer patients without consideration of family history [20,21].

## 3. DDR Role in Pancreatic Cancer

DNA damage is ongoing in living cells as a result of external insults, such as chemicals and ionizing radiation, or innate processes, such as nucleotide mismatches, during replication [22]. DNA damage response (DDR) describes the process of a cell’s recognition of a DNA lesion and triggering of molecular events that result in repair of the abnormality [23]. For the process to advance smoothly and avoidance of daughter cells inheriting the lesion, DDR involves concomitant arrest of the cell cycle [24]. In the case of severe damage, cells may undergo permanent cell cycle arrest (senescence) or programmed cell death (apoptosis) [22].

**Figure 1:**
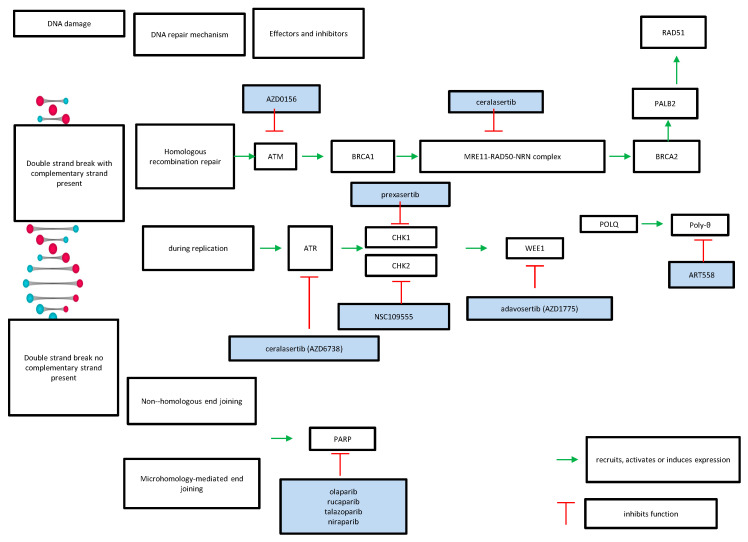
Schematic representation of the repair pathways of double-strand DNA damage and inhibitors discussed in this article.

Different DNA lesions lead to triggering of alternative DDR pathways. Lesions producing single-strand DNA breaks are repaired by base excision repair, bulky adducts in the DNA are repaired by nucleotide excision repair and base mismatches are repaired by the mismatch repair pathway [25]. Double-strand breaks may be repaired by different mechanisms depending on the phase of the cell cycle and presence of a complementary double-strand. If a complementary double DNA strand is present, double-strand breaks may be repaired through HR repair using the intact DNA helix as a template [25]. This process is initiated by kinases ATM and ATR, which recognize the double break and recruit other proteins, such as *BRCA1* and *BRCA2*, *PALB2* and RAD homologous proteins (Figure 1). The cascade starts with kinase ATM recruiting BRCA1 at the DNA damaged site. BRCA1 serves as the docking site for the MRN complex consisting of proteins *MRE11*, *RAD50* and NBN, which create single-strand extensions in the broken site by 5′ end rejections. BRCA2, in co-operation with *PALB2,* helps load *RAD51* onto the single-strand DNA projections, which become able to invade the sister strands and use the homologous strand as a template for production of DNA extensions that are then ligated to repair the break [26]. Kinase ATR senses double breaks during replication and replication fork stalling [22]. ATR inhibits CDK activity, which is initially required for DNA end resection, to promote cell cycle arrest [27]. In the absence of a complementary strand, double-strand breaks are repaired using alternative mechanisms, such as non-homologous end-joining and alternative or microhomology-mediated end-joining, which, in contrast to homologous recombination, are more prone to errors [28,29]. In cancers with *BRCA1*/*BRCA2* mutations or other defects in HR repair, cells are dependent on poly-adenosyl-ribose polymerase (PARP) enzymes to carry repair of their damaged DNA and are thus prone to apoptosis if the enzyme is inhibited by PARP inhibitors, a concept known as synthetic lethality [30]. PARP also plays a role in repair by microhomology-mediated end-joining, which would make *BRCA1* and *BRCA2* defective cells exposed to PARP inhibitors vulnerable to apoptosis [31]. In contrast, cells with no *BRCA1* or *BRCA2* defects are much less sensitive to PARP inhibition. Polymerase theta, encoded by *POLQ* gene, is the polymerase that fills the gaps during microhomology-mediated end-joining and antagonizes homologous recombination by competing with *RAD51* loading [32]. Up-regulation of polymerase theta due to *POLQ* amplification or *POLQ* expression up-regulation, which is observed in cells with *TP53* mutations, such as many pancreatic cancer cells, may lead to homologous recombination defects [33]. Defects in transcription-associated kinase CDK12, which, in conjunction with cyclin K, phosphorylates the carboxyterminal domain of RNA polymerase II and activates transcription, may also lead to homologous recombination dysfunction through down-regulation of genes involved in the process [34].

Detection of defects in HR, besides the presence of oncogenic mutations in BRCA1/BRCA2 and other DDR-involved genes, may be performed with functional assays that detect the results of HR deficiency on the whole genome rather than the causative mutation [35]. A few such assays, including Mychoice HRD (Myriad Genetics Inc., Sault Lake City, UT, USA) and FoundationFocus (Foundation Medicine), have been commercialized and are used clinically [36,37]. Another assay called HRDetect was developed using a logistic regression model trained to identify signatures predictive of the presence of *BRCA1* or *BRCA2* deficiency [38]. An HRD test based on immunohistochemistry instead of genomic signatures has also been developed and detects HR protein RAD51 [39]. RAD51 immunohistochemistry showed high concordance with presence of *BRCA1* and *BRCA2* gene mutations and with the genomic HRD score from the Mychoice HRD assay. Through performance of these assays, it has been reported that the HR defect (as measured by the HRD score) produced by different involved gene mutations is not of the same critical importance. A study that reported on HRD score of pancreatic tumors, for example, showed that the median HRD score of samples with *BRCA1*/*BRCA2* mutations was 44, while the median HRD score of cancers with other HR related mutations was 20 [40].

## 4. Platinum-Based Chemotherapy for *BRCA1*/*BRCA2*-Germline-Mutated Pancreatic Cancers

Due to their underlying molecular lesions, HR-defective cancer cells and tumor-derived xenografts present with decreased ability to repair DNA abnormalities produced by exposure to platinum drugs [41]. In a human pancreatic xenograft study, platinum (and talazoparib) sensitivity was observed in xenografts with biallelic inactivation of *BRCA1* or *BRCA2* [42]. A neo-adjuvant chemotherapy study in patients with germline *BRCA1*/*BRCA2* mutations and borderline resectable pancreatic cancer showed a complete pathologic response rate of 44.4% [43]. Patients with no germline *BRCA1*/*BRCA2* mutations had a 10% complete pathologic response rate in this study and 13% in another study [44]. Complete pathologic responses correlate well with long-term survival [43]. In the adjuvant setting, pancreatic cancer patients with germline *BRCA1* and *BRCA2* mutations who received adjuvant chemotherapy that included platinum drugs had a median survival of 31 months compared with 17.8 months for patients who received non-platinum adjuvant chemotherapy and 9.3 months in patients who had no chemotherapy [45]. In a report of resected pancreatic adenocarcinoma, patients with germline *BRCA1*/*BRCA2* and *PALB2* mutations had better overall survival (OS) than patients without such mutations (median OS 46.6 months versus 23.2 months) [46]. Those patients who had a germline *BRCA1*/*BRCA2* and *PALB2* mutation and received platinum-based perioperative chemotherapy had the longest survival, with median OS not reached at the time of the report [46]. Patients without germline *BRCA1*/*BRCA2* and *PALB2* mutations who received perioperative platinum-based chemotherapy had a median OS of 23.1 months. Patients with mutations in the three genes not receiving perioperative platinum chemotherapy had worse OS than those who received such chemotherapy, although the difference did not reach statistical significance (*p* = 0.07), possibly due to small numbers [46]. Pancreatic cancer patients across stages who were categorized as DDR-deficient based on the presence of germline or somatic mutations in *BRCA1*/*BRCA2* or other DDR gene mutations had better median OS than DDR-proficient patients when exposed to platinums but not if platinum drugs were not part of their treatment [47].

In the advanced stage setting, patients with germline *BRCA1*/*BRCA2* and *PALB2* mutations had an overall response rate to platinum-based chemotherapy of 58% compared to a response rate of 21% in patients without such mutations [48]. In another study, among 58 patients with germline *BRCA1*/*BRCA2* mutations who received first-line platinum-based chemotherapy, 79% had partial responses and an additional 10% had stable disease [49]. In patients with somatic *BRCA1*/*BRCA2* mutations, six of seven (84%) had partial responses to first-line platinum-based chemotherapy in this study [49]. A cohort of 29 advanced pancreatic cancer patients with deleterious germline mutations in *BRCA1*/*BRCA2* or *PALB2* had longer OS (median OS 21.8 months versus 8.1 months) than matched controls without mutations [50]. The difference was driven by the sub-groups treated with platinum-based chemotherapy, while, in the groups not receiving platinum, there was no difference in OS between patients with and without germline *BRCA1*/*BRCA2* or *PALB2* mutations [50]. In a phase 2 study that included stage III and IV pancreatic cancer patients with germline *BRCA1*/*BRCA2* and *PALB2* mutations who were treated with cisplatin and gemcitabine with or without veliparib, response rates in the two arms were 74.1% and 65.2%, which did not differ statistically significantly with addition of the PARP inhibitor [51]. This trial supports the benefit of using platinum-based regimen as an upfront systemic therapy for patients with germline *BRCA1*/*BRCA2*-associated advanced pancreatic cancer as the OS in both arms was superior compared with historical data (median OS 15.5 months with gemcitabine/cisplatin and 16.4 months with gemcitabine/cisplatin/veliparib).

Non-platinum alkylating agent mitomycin C has also been reported to possess efficacy in a few cases of metastatic pancreatic cancer patients with germline *BRCA2* mutations refractory to platinum and olaparib, but prospective data do not exist [52].

## 5. Clinical Trials of PARP Inhibitors in Pancreatic Cancers with Germline *BRCA1*/*BRCA2* Mutations

PARP inhibitors are drugs that inhibit PARP enzymes and constitute the only targeted therapies used in pancreatic cancer indicated for tumors with germline *BRCA1*/*BRCA2* mutations [53]. A benefit of maintenance olaparib versus placebo in prolonging PFS in first-line metastatic pancreatic cancer patients with germline *BRCA1*/*BRCA2* mutations was observed in the Pancreas Cancer Olaparib Ongoing (POLO) phase III randomized double-blind trial [54]. In POLO, patients who had received at least 16 weeks of first-line platinum-based chemotherapy for metastatic pancreatic cancer and had no evidence of disease progression were assigned to olaparib or placebo. Crossover to olaparib was not allowed per study protocol. The primary endpoint of the trial, PFS, was met compared with placebo. Median PFS was 7.4 months with olaparib maintenance versus 3.8 months with placebo (HR: 0.53, 95% CI: 0.35–0.82, *p* = 0.004). A benefit of olaparib regarding time off chemotherapy was observed and is clinically valuable [55]. In addition, a sub-set of patients were long-term survivors. Unfortunately, this phase III trial disclosed no OS benefit from maintenance olaparib (median 19 months versus 19.2 months in the placebo arm, HR: 0.83, 95% CI = 0.56–1.22, *p* = 0.34). Furthermore, the control arm of the trial (assigning patients with stable disease after 16 weeks of induction chemotherapy to placebo) may have been suboptimal compared to the standard practice in many centers, which includes continuation of the same induction chemotherapy until progression or until unacceptable toxicity or other forms of maintenance chemotherapy (e.g., capecitabine/5-fluorouracil or FOLFIRI following induction FOLFIRINOX). Olaparib-induced toxicity (40% of the olaparib arm in the trial experienced grade 3 or higher adverse events, including anemia, fatigue and decreased appetite) and drug cost should also be factored in when the olaparib maintenance strategy is considered for individual pancreatic cancer patients. A similar randomized phase II trial of maintenance olaparib in resected pancreatic cancer and pathogenic *BRCA1*/*BRCA2* or *PALB2* mutations (ECOG-ACRIN EA2192, APOLLO) is enrolling patients with either germline or somatic mutations who have received 3 to 6 months of peri-operative chemotherapy.

A phase II study of olaparib monotherapy in patients with various metastatic malignancies and germline *BRCA1*/*BRCA2* mutations included 23 patients with pancreatic adenocarcinoma [56]. Seventeen patients had *BRCA2* mutations, five patients had *BRCA1* mutations and one patient had both *BRCA1* and *BRCA2* mutations. Patients received a mean of two prior therapies and all but one patient received gemcitabine. Two thirds received prior platinum-based chemotherapy. Response rate was 21.7% (95% CI: 7.5–43.7%) and 35% of patients had stable disease for more than 8 weeks.

A retrospective report of metastatic pancreatic cancer patients with various DDR-related germline mutations who received olaparib included 7 patients with *BRCA1* (*n* = 2) or *BRCA2* (*n* = 5) mutations [57]. Patients had received a median of 2 prior lines of therapy for metastatic disease. Median OS was 24.1 months (range: 9.7–31.8 months).

Rucaparib was tested in a phase II trial in 16 germline *BRCA1 (n= 12)*/*BRCA2* (*n* = 4) mutant pancreatic adenocarcinoma patients and 3 additional patients with somatic *BRCA2* mutations who had received one or two chemotherapy lines [58]. Confirmed response rate was 15.8% (3 of 19 patients) and disease control rate (DCR) was 31.6%. Patients with one prior chemotherapy line had a DCR of 44.4%. Two of the three somatic *BRCA2* mutation patients were among the responders. Loss of heterozygosity (LOH) in *BRCA1*/*BRCA2* loci was evaluable in only two patients and both patients showed LOH for the normal allele [58]. Although the results from this study were encouraging, suggesting a clinical benefit, enrollment was stopped as pre-specified in the protocol because of an insufficient response rate among the first 15 patients.

A phase II trial of maintenance rucaparib in 42 germline pathogenic *BRCA1*/*BRCA2* and *PALB2* mutant (2 patients with somatic *BRCA2* mutations were also included) advanced pancreatic cancer patients who had received platinum-based chemotherapy without evidence of progression disclosed a median PFS of 13.1 months (95% CI: 4.4–21.8 months) and a median OS of 23.5 months (95% CI: 20–27 months) [59]. The response rate was 41.7% (95% CI: 25.5–59.2%). Three of six patients with germline *PALB2* mutations and one of the two patients with somatic *BRCA2* mutations were among the responders.

Combination therapy of PARP inhibitors with immunotherapy treatments are explored in trials in progress. As an example, the SWOG S2001 trial (NCT04548752) compares the combination of olaparib with pembrolizumab versus olaparib alone in maintenance setting in patients with germline *BRCA1*/*BRCA2* mutations.

## 6. PARP Inhibitors for Pancreatic Cancers with Somatic *BRCA1*/*BRCA2* Mutations

Compared with the ample data on PARP inhibitors in pancreatic cancers with germline *BRCA1*/*BRCA2* mutations, data on cases with somatic mutations in the two genes are sparse. A meta-analysis and systematic review that attempted to compare responses to PARP inhibitors between germline and somatic *BRCA1*/*BRCA2* mutations across cancer types concluded that the drugs appear to be equally effective but noted heterogeneity and possible publication bias [60]. Out of eight studies included, the two pancreatic cancer studies that have been discussed in the previous section included a total of four patients, three of whom had a response [58,61]. One of the two studies included in the meta-analysis was in abstract form at the time and in its final version has included an additional patient with a somatic *BRCA2* mutation, and, thus, the final response rate of somatic *BRCA1*/*BRCA2* patients in the two studies combined is 60% (three of five patients) [58,59]. Some of the somatic mutations in *BRCA1*/*BRCA2* (and other DDR-related) genes are mono-allelic or non-pathogenic and thus not producing defects in HR as the normal allele rescues the repair defect. Mouse models of targeted disruption of brca1 have shown that biallelic defects are either embryonic lethal or lead to early cancer-related mortality [62,63]. The specific defects in the two alleles are also important given that different domains of each protein participate in different functions in DDR that could be preserved if the other allele is wild type for the protein domain involved in the respective function. For example, a compound mutant mouse model in which the one *BRCA1* allele has a mutation in the coiled-coil motif of the protein, required for interaction with PALB2, and the other allele has a mutation in the RING domain required for end resection are nearly normal [64]. Consistent with these preclinical observations, in prostate cancer patients with somatic pathogenic frameshift monoallelic *BRCA1* and *BRCA2* mutations in the context of Microsatellite instability, tumors were not sensitive to PARP inhibitors, while they responded to immune checkpoint inhibitors [65]. Interrogation of *BRCA1* and *BRCA2* mutations across cancer types revealed that they were more common in tumors with microsatellite instability compared with microsatellite-stable tumors, but they were in general monoallelic and lacked an association with genome-wide LOH, suggesting that they had no functional repercussions [65]. In contrast, biallelic alterations in genes involved in HR were associated with elevated genomic scar scores, such as elevated HRD scores, and with mutation signature 3, denoting HR insufficiency across cancer types [66]. Thus, the allelic status of somatic mutations in *BRCA1* and *BRCA2* is of therapeutic relevance and will need to be considered in designing studies and evaluating effectiveness of PARP inhibitors and other drugs targeting the pathway. Moreover, the fact that somatic mutations, in contrast to germline mutations, may not be present in all cells of an individual tumor, depending on the timing of their development in the tumor lifetime, may lead to differential sensitivity of individual clones to PARP inhibitors. In these cases, resistance derived from treatment pressure would be expected to arise as clones with no mutations would survive and lead to cancer progression.

## 7. PARP Inhibitors and Other Targeted Therapies for Pancreatic Cancers with Germline or Somatic Mutations in Other DDR-Associated Genes

As discussed in a previous section, besides *BRCA1* and *BRCA2* mutations, mutations in *PALB2*, *ATM*, *ATR*, *BRIP1*, *BARD1* and *CDK12* are observed in low frequencies in pancreatic cancers. These pancreatic cancer cases with defects in other genes of the DDR pathway could be candidates for treatment with PARP inhibitors or other targeted therapies based on dysregulation in DNA repair that these defects cause (Table 3) [67,68].

Mutations in kinase *ATM* are the genetic defects in hereditary cancer-predisposing ataxia–telangiectasia syndrome and underline a sub-set of familial pancreatic cancers [22,69]. The frequency of *ATM* mutations in pancreatic cancers and the critical role of the kinase in DDR and repair could form the basis for therapeutic targeting in pancreatic cancers with such mutations. A theoretical interest in PARP inhibition in *ATM*-mutated cancers stems from the involvement of the kinase in the same HR repair pathway as *BRCA1* and *BRCA2*. Decreased expression of ATM and of the activated form that is phosphorylated at serine 1981 (S1981) are associated with worse survival in pancreatic cancer patients [70]. Defects in ATM function produce increased reliance of cells on the related kinase ATR to prevent replication fork collapse and avoid apoptosis in proliferating cells [71]. In preclinical studies in cells with ATM defects, olaparib induced reversible cell cycle arrest and required addition of an ATR inhibitor to kill these cells [72]. In another study with pancreatic cancer cells treated with gemcitabine in combination with ATR inhibitor ceralasertib (AZD6738), pharmacologic inhibition with the ATM inhibitor AZD0156 or knockout through CRISP-R of ATM led to replication catastrophe and cell death [73]. In cells with reduced but not completely abrogated ATM activity, obtained through siRNA, the combination of gemcitabine and ceralasertib was less effective [73]. Ceralasertib prevented activation of CHK1 kinase induced by gemcitabine abrogating cell cycle arrest [74]. An in vitro and in vivo mouse model of *kras*-driven pancreatic cancer cells with or without concomitant *atm* knockdown revealed significant increase in genomic instability and accumulation of double-strand DNA breaks (as shown by colocalized foci stain for γ-H2AFX and 53BP1) in *atm*-deficient cells and tumors compared with cells and tumors with intact *atm* [75]. In addition, *atm* knockdown promoted early acinar to ductal metaplasia, epithelial to mesenchymal transition and increased metastases in a mouse model [76]. *Atm*-deficient cells were also more sensitive to PARP and ATR inhibitors and to irradiation [75]. Sensitivity of human *ATM*-mutated cells from various cancers to combinations of PARP inhibitors and ATR inhibitors has been observed [77,78]. Mechanistically, PARP inhibition by olaparib causes activation of the G2M checkpoint through the ATR/CHK1 pathway and accumulation of ATM-defective cells in G2M, while addition of ATR inhibitor ceralasertib abrogated the checkpoint and allowed cells with DNA damage to undergo mitosis [79]. This leads to induction of excessive genome instability, micronuclei formation and cell death, a sequence observed with different ATR inhibitors and across cancer types [79,80]. However, the clinical benefit of PARP inhibition in patients with ATM defects or ATM-reduced expression has not been documented. For example, a clinical trial of paclitaxel with or without olaparib in second-line treatment of advanced metastatic gastric cancer examined reduced ATM expression, defined as less than 25% tumor cells staining positive in an immunohistochemistry assay, as a marker of benefit [81]. In the overall population, independent of ATM expression, addition of olaparib did not influence OS. Similarly, patients with negative ATM tumor expression did not benefit from the addition of olaparib to paclitaxel. In a pertinent case report, a patient with metastatic pancreatic cancer and a germline pathogenic ATM mutation progressed on FOLFIRINOX therapy followed by olaparib and subsequently progressed on third-line combination of a PARP and an ATR inhibitor [82]. She obtained a response with nab-paclitaxel/gemcitabine, suggesting that, at least for some patients with these mutations associated with DDR, the expected sensitivity to platinum-based chemotherapy and PARP-based combinations is, disappointingly, not observed. Nevertheless, a systematic examination of ATR inhibitors and combinations with chemotherapy or PARP inhibitors specifically in pancreatic cancer patients with *ATM* mutations has not been performed and needs to be investigated prospectively in a clinical trial.

Kinase WEE1 is a downstream target of ATR and ATM kinases acting on kinase CDK1 to block cell cycle progression at the G2M checkpoint [83,84]. WEE1 is activated by kinases CHK1 and CHK2, which are the direct targets of ATR and ATM following DNA damage [84]. Up-regulation of WEE1 after treatment of pancreatic cancer cells with DNA damaging chemotherapy leads to treatment resistance [85]. Inhibition of WEE1 impairs the G2M checkpoint, leading to mitotic catastrophe [86]. Inhibition of WEE1 has synergistic effects with ATM inhibitors in pancreatic cancer preclinical models in vitro and in vivo [87]. WEE1 inhibitor adavosertib (AZD1775) synergizes with ATM inhibitor AZD0156 in reducing proliferation of pancreatic cancer cells. In addition, the combination down-regulates PD-L1 immune ligand and other immune receptors that are involved in tumor immune evasion [87]. Adavoserib synergizes with ATR inhibitor ceralasertib (AZD6738) in killing cells of various cancer origins [88]. Mechanistically, ATR inhibition prevents feedback activation of ATR produced by WEE1 inhibition. WEE1 inhibitors may also be synergistic with nucleoside analogue chemotherapy drugs, such as gemcitabine, by reducing the ATR and CHK1 activation induced by gemcitabine exposure of pancreatic cancer cells [89]. Several of the discovered WEE1 inhibitors are of clinical grade and a few are in early-phase clinical development, although, currently, few trials have been specific for pancreatic cancer patients and no trials have used molecular markers of DNA damage for patient selection. A phase Ib dose escalation trial of adavosertib in combination with gemcitabine and radiation therapy in locally advanced pancreatic cancer patients showed a median PFS of 9.4 months (95% CI: 8 months to 9.9 months) and a median OS of 21.7 months (95% CI: 16.7 months to 24.8 months) [90]. The recommended phase II dose of adavosertib was 150 mg per day and, at this dose, pharmacodynamic studies showed inhibition of WEE1 target CDK1 kinase. The observed survival outcomes were deemed encouraging compared with historical results with gemcitabine and radiation therapy in this population of patients [90]. Perhaps counter-intuitively, the preclinical study that led to this clinical trial had suggested that the benefit of the triple adavosertib/gemcitabine/radiation combination is mostly observed in HR proficient cells with wild type *BRCA2*, while *BRCA2* mutant cells derived smaller benefit [91]. An explanation of the results with this particular combination may relate to the synergistic effect of radiation therapy and WEE1 inhibition in eliciting an immune response, the former activating the STING pathway while the latter down-regulates inhibitory immune ligands. HR proficient cells are, then, less prone to immune escape as they are genomically stable compared with HR-deficient cells.

CHK2 kinase acts downstream of kinase ATM to induce G1 phase arrest in untransformed cells following double-strand DNA breaks [92]. Sustained activity of CHK2 is required for maintenance of the checkpoint, while the activity of ATM, or homologous kinases ATR and DNA-PKcs, is not required after checkpoint induction. In pancreatic cancer cell lines, CHK2 inhibitor NSC109555 displays a synergistic inhibitory effect with gemcitibine [93]. Similarly, synergistic inhibition was observed when CHK2 was knocked down through siRNA. Homologous kinase CHK1 plays a similar role in activating cell cycle arrest after activation by kinase ATR. CHK1 inhibition by inhibitor prexasertib was also shown to synergistically inhibit pancreatic cancer cells together with gemcitabine and the antimetabolite S-1 [94]. Although the clinical development of CHK1 and CHK2 inhibitors has started with phase I and II trials as monotherapy or in combination with other drugs, no trials specifically in pancreatic cancers have been currently registered.

DNA polymerase theta encoded by *POLQ* is involved in one of the double-strand breaks repair mechanisms called polymerase-theta-mediated end-joining (TMEJ, alternatively named microhomology-mediated end-joining) [32]. The enzyme has both polymerase and helicase activity [95]. It is also involved in other DNA repair mechanisms, including translesion synthesis and base excision repair, helped by lyase activity detected in the polymerase domain [96]. Polymerase theta up-regulation is observed in cancer tissues. Moreover, synthetic lethality of *POLQ* with *BRCA1* and *BRCA2* and other HR genes has been described [97,98]. A CRISP-R-based screen discovered further synthetic lethalities of *POLQ* with knockdown of genes involved in other DNA repair pathways, including base excision repair, nucleotide excision repair and mismatch repair [99]. Some of these synthetic lethal associations have been validated mostly in models other than pancreatic cancer, and whether they are valid in pancreatic cancer awaits further investigation [32]. Cells with TP53 mutations, which frequently occur in pancreatic cancer, significantly over-express POLQ [33]. This over-expression is associated with promotion of DNA repair through TMEJ and preserved cell viability following exposure to radiomimetic drug neocarzinostatin. Polymerase theta inhibitors have been discovered and are in development [100,101]. Novel inhibitor ART558 has entered phase I/II clinical trials and will be investigated alone or in combination with PARP inhibitors talazoparib and niraparib (NCT04991480). The role of polymerase theta inhibition in pancreatic cancers with *BRCA1*/*BRCA2* and non-BRCA HR defects and possibly defects in other repair pathways could be the logical step for investigation in future trials.

## 8. Conclusions

Pancreatic cancers with alterations in the machinery that detects and repairs double-strand DNA breaks represent a group of cancers with potential to be targeted therapeutically with inhibitors of proteins involved in the repair process. Individual abnormalities and the molecular micro-environment of the tumor will have to be taken into consideration for rational personalized drug development. Combination therapies that would prevent development of resistance associated with monotherapy will most certainly be required for optimal and long-lasting results to improve outcomes in one of the most lethal cancers. A common theme in the mechanism of synergy of ATR, WEE1 and CHK1/2 inhibitors with other drugs, such as PARP inhibitors and chemotherapy, is abrogation of the cell cycle checkpoint induced by these various drugs and mitotic catastrophe promotion [74,75,89]. This mechanism may be explored in broader therapeutic scenarios, such as various immunotherapy approaches. For example, ceralasertib was shown to synergize with an adenovirus-based oncolytic immunotherapy in a mouse model of glioma in vitro and in vivo [102]. In addition, even though mismatch repair deficiency is rare in pancreatic adenocarcinomas, other molecular lesions, such as ARID1A mutations, may confer immune checkpoint inhibitor sensitivity and could become the basis for synergistic combinations [103]. Discovery of rational combinations in preclinical models is a first step [104]. Subsequent clinical trials, many currently ongoing, will confirm pre-clinical observations and will determine whether these combinations are feasible from the adverse effect point of view.

## Figures and Tables

**Table 1:** Alterations in DDR-related genes in pancreatic cancers from TCGA and MSK-IMPACT cohorts. NA: not available.

Gene	TCGA All Alterations (*n* = 184 Profiled)	Amplifications	Mutations	(Likely) Oncogenic Mutations	MSK-IMPACT All Alterations (*n* = 383 Profiled)	Amplifications	Mutations	(Likely) Oncogenic Mutations
*BRCA1*	6 (3.3%)	4 (2.2%)	2 (1.1%)	0	5 (1.3%)	0	4 (1%)	2 (0.5%)
*BRCA2*	2 (1.1%)	0	2 (1.1%)	2 (1.1%)	13 (3.4%)	0	13 (3.4%)	10 (2.6%)
*PALB2*	1 (0.5%)	0	1 (0.5%)	0	2 (0.5%)	0	2 (0.5%)	1 (0.3%)
*RAD51*	1 (0.5%)	1 (0.5%)	0	0	0			
*RAD51B*	0				0			
*RAD51C*	1 (0.5%)	0	1 (0.5%)	1 (0.5%)	0			
*RAD51D*	4 (2.2%)	4 (2.2%)	0	0	1 (0.3%)	0	0	0
*RAD50*	3 (1.6%)	2 (1.1%)	0	0	3 (0.8%)	0	2 (0.5%)	0
*XRCC2*	2 (1.1%)	2 (1.1%)	0	0	1 (0.3%)	0	1 (0.3%)	0
*ATM*	8 (4.3%)	0	8 (4.3%)	4 (2.2%)	11 (2.9%)	0	11 (2.9%)	5 (1.3%)
*ATR*	5 (2.7%)	1 (0.5%)	4 (2.2%)	2 (1.1%)	1 (0.3%)	0	1 (0.3%)	0
*BRIP1*	5 (2.7%)	4 (2.2%)	1 (0.5%)	0	4 (1%)	2 (0.5%)	2 (0.5%)	1 (0.3%)
*NBN*	8 (4.3%)	8 (4.3%)	0	0	3 (0.8%)	2 (0.5%)	1 (0.3%)	0
*MRE11*	1 (0.5%)	0	1 (0.5%)	0	0			
*CHEK1*	0				2 (0.5%)	0	1 (0.3%)	0
*CHEK2*	1 (0.5%)	0	1 (0.5%)	0	0			
*BARD1*	5 (2.7%)	3 (1.6%)	2 (1.1%)	0	3 (0.8%)	1 (0.3%)	3 (0.8%)	2 (0.5%)
*WEE1*	1 (0.5%)	1 (0.5%)	0	0	NA			
*PRKDC*	9 (4.9%)	6 (3.3%)	3 (2%)	0	NA			
*POLQ*	3 (1.6%)	2 (1.1%)	1 (0.5%)	0	NA			
*CDK12*	9 (4.9%)	8 (4.3%)	1 (0.5%)	0	8 (2.1%)	6 (1.6%)	2 (0.5%)	1 (0.3%)
Total (non-overlapping)	42 (22.8%)	29 (15.8)	15 (8.2%)	9 (4.9)	46 (12%)	11 (2.9%)	37 (9.7%)	20 (5.2%)

**Table 2:** Alterations in DDR-related genes in pancreatic cancers from Chinese Origene and project GENIE pancreatic cancer cohorts. NA: not available.

Gene	Chinese All Alterations (*n* = 461 Profiled)	Amplifications	Mutations	(Likely) Oncogenic Mutations	Project GENIE All Alterations (*n* = 5874 Profiled)	Amplifications	Mutations	(Likely) Oncogenic Mutations
*BRCA1*	5 (1.1%)	0	5 (1.1%)	1 (0.2%)	110 (2%)	1 (0.02%)	107 (1.9%)	50 (1%)
*BRCA2*	8 (1.7%)	0	8 (1.7%)	5 (1.1%)	242 (4.5%)	7 (0.1%)	237 (4.5%)	143 (2.8%)
*PALB2*	7 (1.5%)	0	7 (1.5%)	3 (0.7%)	75 (1.4%)	1 (0.02%)	72 (1.4%)	35 (0.6%)
*RAD51*	1 (0.2%)	0	0		7 (0.2%)	0	7 (0.1%)	2 (0.04%)
*RAD51B*	3 (0.7%)	0	2 (0.4%)	0	6 (0.2%)	1 (0.02%)	6 (0.1%)	2 (0.04%)
*RAD51C*	1 (0.2%)	0	1 (0.2%)	1 (0.2%)	17 (0.4%)	1 (0.02%)	13 (0.2%)	2 (0.04%)
*RAD51D*	1 (0.2%)	0	1 (0.2%)	0	9 (0.2%)	4 (0.08%)	4 (0.08%)	2 (0.04%)
*RAD50*	6 (1.3%)	0	6 (1.3%)	3 (0.7%)	41 (0.9%)	0	41 (0.9%)	14 (0.3%)
*XRCC2*	2 (0.4%)	0	2 (0.4%)	1 (0.2%)	15 (0.3%)	0	15 (0.2%)	3 (0.1%)
*ATM*	21 (5%)	0	21 (5%)	16 (3.5%)	251 (4.5%)	0	271 (5.2%)	171 (3.5%)
*ATR*	6 (1.3%)	0	6 (1.3%)	3 (0.7%)	97 (1.9%)	1 (0.02%)	96 (1.9%)	28 (0.6%)
*BRIP1*	2 (0.4%)	0	2 (0.4%)	0	62 (1.2%)	21 (0.4%)	41 (0.8%)	18 (0.3%)
*NBN*	0				38 (0.7%)	5 (0.1%)	35 (0.7%)	14 (0.3%)
*MRE11*	1 (0.2%)	0	1 (0.2%)	1 (0.2%)	15 (0.3%)	1 (0.02%)	14 (0.3%)	2 (0.04%)
*CHEK1*	1 (0.2%)	0	1 (0.2%)	0	15 (0.3%)	0	15 (0.3%)	3 (0.06%)
*CHEK2*	0				34 (0.7%)	0	34 (0.6%)	13 (0.2%)
*BARD1*	2 (0.4%)	0	2 (0.4%)	0	50 (1%)	1 (0.02%)	48 (1%)	8 (0.1%)
*WEE1*	1 (0.2%)	0	1 (0.2%)	0	NA	NA	NA	
*PRKDC*	10 (2.2%)	1 (0.2%)	9 (2%)	0	100 (4.1%)	5 (0.1%)	94 (4%)	NA
*POLQ*	0				46 (2.9%)	0	46 (2.9%)	NA
*CDK12*	5 (1.1%)	5 (1.1%)	0		79 (1.6%)	34 (0.7%)	41 (0.8%)	8 (0.1%)
Total non-overlapping	76 (16.5)	6 (1.3)	71 (15.4)	30 (6.5)	1062 (18.1)	61 (1.04)	940 (16)	336 (5.7)

**Table 3:** Inhibitors targeting DNA damage response.

Repair Pathway	Targeted Gene	Inhibitor
**homologous recombination**	*ATR*	ceralasertib
	*ATM*	AZD0256
	*CHK2*	NSC109555
	*CHK1*	prexasertib
	*WEE1*	adavosertib
	*POLQ*	ART558
**non-homologous end-joining or microhomology-mediated end-joining**	*PARP*	olaparib rucaparib talazoparib niraparib

## Data Availability

There are no data available beyond the data presented in the manuscript.
